# Comparative Transcriptome Analysis Elucidates the Desiccation Stress Adaptation in *Sargassum muticum*

**DOI:** 10.3390/genes16050587

**Published:** 2025-05-16

**Authors:** Wei Cao, Mingyi Zhang, Nan Wu, Yanxin Zheng, Xiaodong Li, Haiying Han, Tao Yu, Zhongxun Wu, Pei Qu, Bo Li

**Affiliations:** 1 Island and Reef Fishery Research Center, Changdao Enhancement and Experiment Station, Chinese Academy of Fishery Science, Yantai 264000, China; caowei1996@foxmail.com (W.C.); 13050348400@163.com (M.Z.); jmscxywn@163.com (N.W.); cd-zzz@126.com (H.H.); cdyutao@126.com (T.Y.); 2 Observation and Research Station of Bohai Strait Eco-Corridor, MNR, Qingdao 266000, China; qupei@fio.org.cn; 3 Key Laboratory of Breeding Biotechnology and Sustainable Aquaculture (CAS), Institute of Oceanology, Chinese Academy of Sciences, Qingdao 266000, China; xdli@qdio.ac.cn; 4 Laboratory for Marine Biology and Biotechnology, Qingdao Marine Science and Technology Center, Qingdao 266000, China; 5 National Ocean Park Administration Center of Changdao, Yantai 264000, China; cdwuzx@163.com

**Keywords:** *Sargassum muticum*, brown seaweed, desiccation stress, RNA-seq

## Abstract

Background/Objectives: Desiccation profoundly influences the distribution and abundance of intertidal seaweeds, necessitating robust molecular adaptations. *Sargassum muticum* is a brown seaweed inhabiting intertidal rocky substrates. During low tides, this species undergoes periodic aerial exposure. Such environmental conditions necessitate robust physiological mechanisms to mitigate desiccation stress. Yet, the molecular basis of this adaptation remains poorly understood. Methods: To investigate desiccation-responsive genes and elucidate the underlying mechanisms of adaptation, we exposed *S. muticum* to 6 h of controlled desiccation stress in sterilized ceramic trays, simulating natural tidal conditions, and performed comparative transcriptome analysis using RNA-seq on the Illumina NovaSeq 6000 platform. Results: High-quality sequencing identified 66,192 unigenes, with 1990 differentially expressed genes (1399 upregulated and 591 downregulated). These differentially expressed genes (DEGs) were categorized into regulatory genes—including mitogen-activated protein kinase (*MAPK*), calmodulin, elongation factor, and serine/threonine-protein kinase—and functional genes, such as heat shock protein family members (*HSP20*, *HSP40*, and *HSP70*), tubulin (*TUBA* and *TUBB*), and endoplasmic reticulum homeostasis-related genes (protein disulfide-isomerase A6, calreticulin, and calnexin). Gene Ontology (GO) enrichment highlighted upregulated DEGs in metabolic processes like glutathione metabolism, critical for oxidative stress mitigation, while downregulated genes were linked to transport functions, such as ammonium transport, suggesting reduced nutrient uptake during dehydration. KEGG pathway analysis revealed significant enrichment in “protein processing in endoplasmic reticulum” and “MAPK signaling pathway-plant”, implicating endoplasmic reticulum stress response and conserved signaling cascades in desiccation adaptation. Validation via qRT-PCR confirmed consistent expression trends for key genes, reinforcing the reliability of transcriptomic data. Conclusions: These findings suggest that *S. muticum* undergoes extensive biological adjustments to mitigate desiccation stress, highlighting candidate pathways for future investigations into recovery and tolerance mechanisms.

## 1. Introduction

Water stress significantly impacts plant growth and productivity by disrupting cellular homeostasis, impairing photosynthesis, and reducing biomass accumulation [1]. Drought triggers a cascade of physiological and biochemical responses in plants, including stomatal closure, osmotic adjustment, and the accumulation of reactive oxygen species (ROS), which collectively aim to mitigate water loss and oxidative damage [2]. These adaptive mechanisms operate at both cellular and molecular levels, with plants synthesizing osmolytes such as proline, glycine betaine, and soluble sugars to maintain turgor pressure and stabilize macromolecules under dehydrating conditions [3]. These stresses modulate gene expression, inducing or repressing genes with diverse functions [4,5,6]. Notably, the introduction of stress-inducible genes via genetic engineering has enhanced plant stress tolerance [7,8]. Elucidating the functions of these genes is critical for advancing plant stress response mechanisms, potentially enabling genetic interventions to bolster crop resilience. However, the functional characterization of drought-responsive genes remains incomplete, limiting the translation of molecular insights into practical applications.

Intertidal marine macroalgae, vital primary producers at the base of the marine food chain, serve fundamental ecological roles in aquatic ecosystems [9,10]. They provide habitat complexity, stabilize substrates, and support trophic interactions. Intertidal ecosystems are dynamic environments characterized by extreme fluctuations in abiotic conditions, including temperature, salinity, and water availability. These organisms experience periodic desiccation due to daily tidal fluctuations, necessitating adaptations to withstand significant intracellular water loss [11]. Desiccation poses a critical challenge for marine macroalgae during low tides, as prolonged aerial exposure disrupts cellular homeostasis and induces oxidative damage [10]. While terrestrial plants have evolved specialized structures (e.g., stomata and cuticles) and hormonal pathways (e.g., abscisic acid signaling) to regulate water loss, intertidal seaweeds lack specialized tissues (e.g., roots or stomata) for water loss regulation, relying instead on rapid cellular adjustments [9,10,11]. Desiccation profoundly influences the distribution and abundance of intertidal seaweeds [10]. While molecular insights into drought responses primarily derive from studies on higher green plants under dehydrating conditions [3,6,12,13], research on desiccation tolerance in intertidal macroalgae has largely focused on reactive oxygen species [14]. Understanding these mechanisms is not only pivotal for deciphering ecological resilience but also holds biotechnological potential for improving stress tolerance in economically important algae.

*Sargassum muticum*, a brown seaweed prevalent in cold and warm–temperate waters, including China’s northern coast, has garnered attention for its remarkable invasive success across diverse biogeographic regions, from subarctic to subtropical zones, and its dual role as a keystone species in native habitats and a prolific invader in non-native ecosystems. In its native range along the western Pacific coast, this brown seaweed forms extensive underwater forests that stabilize sediments, reduce hydrodynamic forces, and provide critical nursery grounds for commercially important fish and invertebrates. These ecosystems are biodiversity hotspots, supporting complex trophic interactions that underpin local fisheries. This species has attained unprecedented invasive success among marine macrophytes, as evidenced by its expansive non-indigenous distribution extending from subarctic (Alaska) to subtropical (Baja California) latitudes along the eastern Pacific coasts, while colonizing biogeographic realms ranging from Scandinavian coasts (Norway) to northwest African shores (Morocco) in the eastern Atlantic basin [15]. Notably, the Mediterranean-ecosystem-dominated introduced range exceeds by orders of magnitude its endemic distribution confined to western Pacific coastal zones between southern Russian and Chinese territories. However, this success also raises concerns about its impact on native biodiversity. For instance, in the northeastern Atlantic, *S. muticum* forms dense canopies that outcompete native kelp species, altering habitat structure and trophic dynamics [15]. These observations collectively indicate that the species has the capacity to establish populations across a wide range of environmental regimes through the adaptive regulation of its physiological, developmental, and phenological processes. The brown seaweed ability to colonize intertidal and subtidal habitats underscores its ecological plasticity. In addition, it is ecologically significant as a dominant species in seaweed beds, providing spawning, nursery, and feeding grounds for marine organisms in shallow sublittoral zones [16,17]. It is also valued for alginate production, heavy metal biosorption [18,19,20], and as feed in holothurian and abalone aquaculture in China [17,21]. Furthermore, its adaptability to diverse environments makes it a candidate for constructing artificial seaweed beds [21]. As an intertidal species, *S. muticum* faces periodic air exposure during low tides, leading to water loss. Consequently, it has evolved mechanisms to tolerate desiccation stress. Despite its ecological and economic significance, the genetic basis of its stress signaling pathways and desiccation stress adaptation remains poorly understood, particularly regarding desiccation adaptation—a key determinant of its intertidal zonation.

Recent advances in transcriptomics have enabled genome-wide explorations of stress responses in non-model organisms, but such studies on *Sargassum* spp. are sparse. A notable gap exists in understanding how this genus coordinates stress perception, signaling, and effector gene activation—knowledge that could inform conservation strategies for native ecosystems and enhance aquaculture practices.

This study aims to bridge this gap by employing comparative transcriptomics to identify desiccation-responsive genes in *S. muticum*. By simulating natural tidal emersion and analyzing differential gene expression, we seek to unravel the regulatory and functional networks that underpin its desiccation adaptation. Our findings not only contribute to the broader understanding of algal stress biology but also provide candidate genes for biotechnological applications. The genes highlighted in this study may contribute to desiccation stress adaptation and provide a foundation for future research into the molecular mechanisms underlying stress resilience in *Sargassum*.

## 2. Materials and Methods

### 2.1. Sample Collection and Desiccation Stress

The brown seaweed was collected from the intertidal zone of Sunjia Village, Changdao, Yantai City, Shandong Province (37.925569° N, 120.761500° E), and transported to the laboratory at Changdao Enhancement and Experiment Station. Upon arrival at the laboratory, the samples were rinsed with filtered seawater to remove epiphytes and debris. Acclimation was conducted in a climate-controlled chamber (21 °C; 3500 lx light intensity; 12 h:12 h light–dark cycle) for five days to standardize physiological states prior to experimentation. After acclimation, six well-grown brown seaweed specimens were selected; three were maintained under acclimation conditions as the control group (labeled Group A), while the other three were placed in dry, sterilized ceramic trays for desiccation stress lasting 6 h, as the treatment group (labeled Group B). The 6 h desiccation period was chosen based on the natural tidal cycle, as *S*. *muticum* typically experiences up to 6 h of aerial exposure during low tides in its mid-intertidal habitat. After the stress treatment, surface water was removed from the algal bodies of both groups using absorbent paper. Portions of the axes and blades were immediately excised, snap-frozen in liquid nitrogen, and stored at −80 °C for subsequent use.

### 2.2. RNA Isolation and Library Preparation

Total RNA was extracted using TRIzol reagent (Thermo Scientific, Waltham, MA, USA) according to the manufacturer’s protocol. RNA purity and quantification were assessed using a NanoDrop 2000 spectrophotometer (Thermo Scientific, Waltham, MA, USA). RNA integrity was evaluated with the Agilent 2100 Bioanalyzer (Agilent Technologies, Santa Clara, CA, USA). Libraries were then constructed using the VAHTS Universal V6 RNA-seq Library Prep Kit (Illumina, San Diego, CA, USA) for Illumina following the manufacturer’s instructions. The libraries were sequenced using the Illumina Novaseq 6000 platform (Illumina, San Diego, CA, USA), generating 150 bp paired-end reads. Transcriptome sequencing and analysis were performed by OE Biotech Co., Ltd. (Shanghai, China).

### 2.3. RNA-seq Data Analysis

To ensure robust transcriptome assembly, multiple quality control steps were implemented. Raw FASTQ data were first processed using Trimmomatic (v0.40) [22] to remove reads containing poly-N sequences and low-quality reads, yielding clean reads. Cleaned sequencing reads were processed through a sequential assembly strategy comprising (1) contig construction and (2) Trinity based (v2.4) [23] de novo transcript assembly in paired-end mode. To minimize redundancy, subsequent transcript filtering applied thresholds based on sequence identity and maximum length criteria, retaining the longest isoform per cluster for downstream analyses as unigenes.

Functional annotation integrated systematic database queries against NCBI non-redundant (NR), Swiss-Prot, eggNOG, and eukaryotic KOG databases via DIAMOND (v5.0.2) [24] alignments (e-value < 1 × 10^−5^) with maximum sensitivity settings. NR annotations adopted best reciprocal BLAST (2.6.0+) matches based on sequence similarity thresholds, followed by domain identification via Pfam and structural classification. KEGG pathway mapping [25] elucidated metabolic networks, while Gene Ontology (GO) categorization was derived from established Swiss-Prot to GO associations.

Gene expression levels (FPKM) were calculated using Bowtie2 (v2.5.4) [26] to align reads to unigenes and eXpress (V5.1.0) [27] to quantify expression. Differentially expressed unigenes (DEGs) between groups were identified using DESeq2 (v3.22) [28] in order to calculate the fold of difference and NB (negative binomial distribution test) was used to test the significance of difference, and, finally, the default filter conditions for DEGs were *q* < 0.05 and fold-Change > 2 or fold-Change < 0.5. The hierarchical clustering of DEGs was performed in R 3.2.0 to visualize expression patterns across groups and samples. The GO and KEGG enrichment analyses of DEGs were conducted using R based (v3.2.0) on a hypergeometric distribution, with significant terms illustrated in column and bubble diagrams generated in R 3.2.0.

### 2.4. Validation of DEGs by qRT-PCR

To verify the sequencing results, ten desiccation adaptation candidate genes (seven upregulated and three downregulated) were selected for qRT-PCR validation, with the actin gene being set as the internal reference gene. Transcriptome and qRT-PCR use the same RNA. Firstly, approximately 1 μg total RNA was reversely transcribed into cDNA using HiScript III RT Super Mix for qPCR (Vazyme, Nanjing, China). Secondly, specific primers of qRT-PCR were designed using NCBI Primer 3-blast (Table 1). qRT-PCR was carried out in the CFX Connect Real-Time system (Bio-Rad, Hercules, CA, USA) using the ChamQ SYBR Color qPCR Master Mix (Vazyme, Nanjing, China) following the manufacturer’s instructions. The reactions comprised 2 μL of cDNA, 0.4 μL each of forward and reverse primers, 10 μL mL of ChamQ SYBR Color qPCR Master Mix, and 7.2 μL water. The cycling parameters were as follows: 95 °C for 30 s, followed by 40 cycles at 95 °C for 10 s and 60 °C for 30 s. Finally, the relative expressions of target genes were calculated by the 2^−ΔΔCt^ method. To minimize technical variability during qRT-PCR, three technical replicates per sample were analyzed. Using Graphpad Prism 8.0 software, a histogram analysis was performed using data of qRT-PCR and RNA-seq.

## 3. Results

### 3.1. Transcriptome Sequencing and Unigene Assembly

To comprehensively analyze the transcriptomic landscape and identify dehydration-responsive expression patterns in *S. muticum*, we established six cDNA libraries through mRNA extraction from experimental specimens subjected to 0 h (control) and 6 h desiccation exposures. This experimental design incorporated triplicate biological replicates for each treatment timepoint. Subsequent high-throughput sequencing was conducted individually for each library using the Illumina platform. After data trimming and filtering, approximately 6 Gb of clean data were obtained from each library, totaling 37.91 Gb. Specifically, 123.28 million clean reads were generated, with an average Q30 value of 92.92% (Table 2), indicating that 92.92% of the bases had a quality score of 30 or higher. De novo assembly yielded 66,192 unigenes with an N50 length of 1916 bp. The reliability of downstream transcriptome analytical outcomes is guaranteed by the superior quality metrics obtained from sequencing data.

### 3.2. Annotation of Unigenes

Subsequently, all these unigenes were annotated with seven databases, namely, NR, Pfam, eggNOG, KOG, Swissprot, GO, and KEGG; of these, 36,687 (55.43%) were annotated in the NR database, 25,306 (38.23%) in the Pfam database, 25,060 (37.86%) in the eggNOG database, 20,422 (30.85%) in the KOG database, 19,016 (28.73%) in the Swissprot database, 17,939 (27.10%) in the GO database, and 9558 (14.44%) in the KEGG database, whereas 6969 (10.53%) were annotated in all databases (Table 3). This multi-database approach enhances functional reliability and provides a foundation for probing the molecular mechanisms underlying desiccation adaptation.

### 3.3. Differentially Expressed Gene Analysis

**Figure 1:**
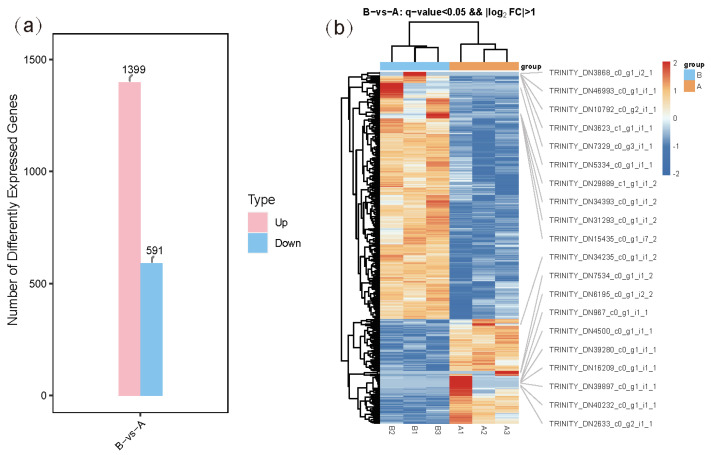
Statistics of DEGs (**a**) and heatmap of all DEGs (**b**).

The differentially expressed genes (DEGs) between the two groups were identified based on gene expression levels in each sample. In group B compared to group A, a total of 1990 DEGs were detected, with 1399 upregulated and 591 downregulated (Figure 1). These findings indicate that desiccation stress significantly altered gene expression. The heatmap analysis confirmed consistent DEG expression patterns across the three replicates in each group (Figure 1). Functional annotations from databases such as NR, Swiss-Prot, and KEGG revealed that upregulated genes included tubulin (*TUBA* and *TUBB*), heat shock proteins (*HSP20*, *HSP40*, and *HSP70*), serine/threonine-protein kinase, protein disulfide-isomerase A6, calreticulin, calnexin, and elongation factor 1-alpha, while downregulated genes comprised ATP-binding cassette transporters and glyceraldehyde 3-phosphate dehydrogenase (Table S1). These DEGs were classified into two main categories: regulatory genes and functional genes.

### 3.4. GO Enrichment and KEGG Pathway Enrichment Analyses of DEGs

**Figure 2:**
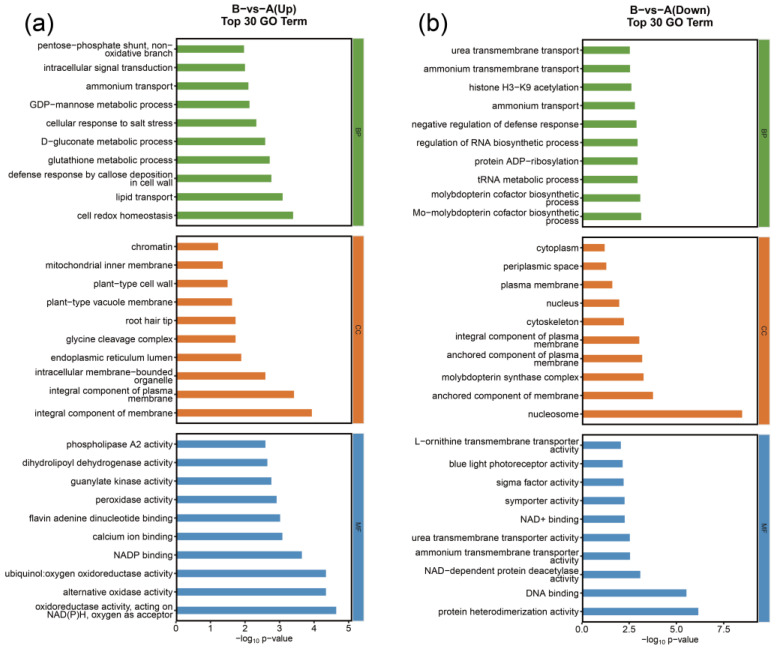
Gene Ontology (GO) enrichment of upregulated DEGs (**a**) and downregulated DEGs (**b**). The bar chart shows the number of DEGs belonging to the top 10 categories (*X*-axis) in the “biological process” (green), “cellular component” (orange), and “molecular function” (blue) GO domains.

To explore the main biological functions and metabolic pathways of DEGs under desiccation stress, GO and KEGG enrichment analyses were performed, respectively. The GO enrichment analysis of DEGs showed that the upregulated genes were mainly enriched in metabolic processes, including glutathione metabolic process (GO:0006749), D-gluconate metabolic process (GO:0019521), GDP-mannose metabolic process (GO:0019673), mitochondrial inner membrane (GO:0005743), and peroxidase activity (GO:0004601). The downregulated genes were involved in transport, including ammonium transport (GO:0015696), ammonium transmembrane transport (GO:0072488), urea transmembrane transport (GO:0071918), plasma membrane (GO:0005886), and ammonium transmembrane transporter activity (GO:0008519) (Figure 2).

**Figure 3:**
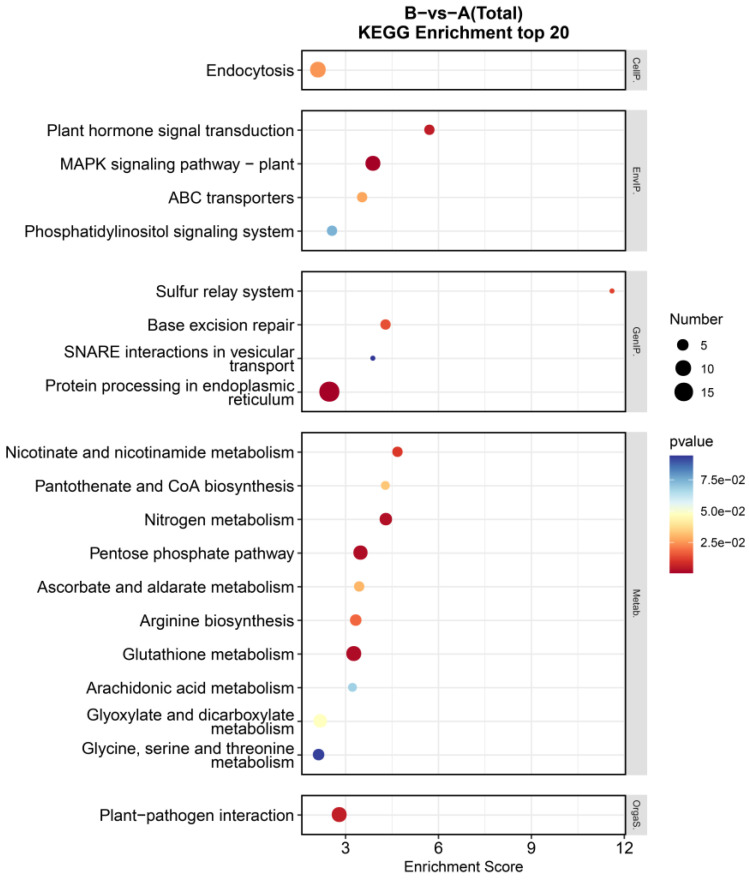
KEGG enrichment analysis for DEGs.

The KEGG enrichment analyses were carried out to explore the signaling pathways associated with DEGs induced by desiccation stress. In total, 1990 DEGs were enriched in 77 pathways (Table S2). Through the analysis of the top 20 pathway maps enriched (Figure 3), we found that these DEGs were mainly involved in their own metabolic processes, including pentose phosphate pathway (ko00030), ascorbate and aldarate metabolism (ko00053), and glyoxylate and dicarboxylate metabolism (ko00630) in carbohydrate metabolism; glutathione metabolism (ko00480) and arginine biosynthesis (ko00220) in amino acid metabolism; and nicotinate and nicotinamide metabolism (ko00760) and pantothenate and CoA biosynthesis (ko00770) in metabolism of cofactors and vitamins. Furthermore, protein processing in endoplasmic reticulum (ko04141) was found to be the most significantly affected pathway. The results from these pathway analyses showed that the processes of maintaining protein homeostasis play important roles in the protection of brown seaweed against desiccation stress. This process mainly elevated the expression of HSP family members (*HSPs*) and endoplasmic reticulum homeostasis-related genes (protein disulfide-isomerase A6, calreticulin, and calnexin) (Table S3). Within the endoplasmic reticulum protein quality control pathway, calreticulin (*CALR*) and calnexin (*CANX*) function as lectin-binding chaperones that operate at the upstream regulatory level through their carbohydrate recognition domains. These molecular chaperones coordinate the initial stages of glycoprotein processing through substrate recognition and retention mechanisms. At the intermediate stage of protein maturation, protein disulfide-isomerase A6 (*PDIA6*) serves as a crucial folding catalyst that facilitates critical processes including proteolytic regulation, folding dynamics, chaperone interactions, and degradation pathways. This oxidoreductase enzyme mediates disulfide bond formation and isomerization through its thioredoxin-like domains, ensuring proper tertiary structure acquisition. The terminal quality assurance phase is primarily mediated by heat shock proteins (*HSPs*) that constitute a sophisticated surveillance network. These molecular chaperones execute stringent quality control through repeated folding attempts and ultimately target irreparably misfolded proteins for endoplasmic reticulum-associated degradation via the ubiquitin–proteasome system. Notably, another interesting pathway, “MAPK signaling pathway-plant” (ko04016), was found, which was also the second most significantly enriched pathway. This pathway was enriched for nine unigenes, including mitogen-activated protein kinase (*MAPK1_2* and *MAPK6*), calmodulin (*CALM*), and serine/threonine-protein kinase (*SNRK2* and *CTR1*) (Table S4). *SNRK2* and *CTR1* function upstream in the signaling cascade, while *MAPK1_2* and *MAPK6* operate downstream, collectively regulating abscisic acid (ABA)-mediated stress adaptation responses in plants. *CALM* is an important Ca^2^⁺ sensor that transmits these signals to maintain the homeostacis of reactive oxygen species.

### 3.5. Validation of Gene Expression by qRT-PCR

**Figure 4:**
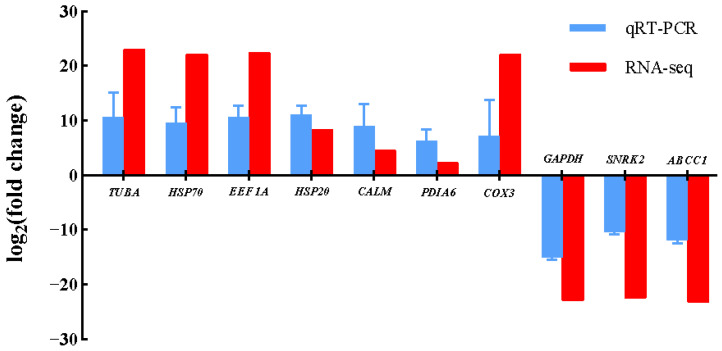
Validation of the RNA-seq data by qRT-PCR. The qRT-PCR data presented are the means ± SD of three biological replicates, and each measurement was repeated three times.

To validate the reliability of the transcriptomic data, ten candidate genes, seven upregulated and three downregulated, were selected for qRT-PCR analysis. As shown in Figure 4, the qRT-PCR analysis of the ten selected genes revealed expression patterns consistent with the transcriptome data, confirming the reliability of the RNA-seq results.

## 4. Discussion

Intertidal seaweeds, subjected to recurrent desiccation during tidal emersion, must rapidly activate molecular and physiological adaptations to survive cellular dehydration. *S*. *muticum*, a sessile species in the intertidal zone, undergoes water loss during low tides, demonstrating high resilience to desiccation stress [1]. However, the molecular mechanisms underpinning this response remain poorly elucidated. To identify genes associated with desiccation stress tolerance in brown seaweed, our transcriptomic analysis under controlled desiccation revealed a robust reprogramming of gene expression, characterized by the differential regulation of 1990 genes. The functional analysis of differentially expressed genes (DEGs) categorized them into two groups: regulatory genes, including mitogen-activated protein kinase, calmodulin, elongation factor, and serine/threonine-protein kinase, and functional genes, such as heat shock proteins (*HSPs*), tubulin, and endoplasmic reticulum homeostasis-related genes. These results indicate substantial gene expression adjustments in *S. muticum* under desiccation stress, suggesting a multifaceted response involving coordinated metabolic and signaling pathways to mitigate its adverse effects.

As global temperatures rise and intertidal zones experience increased desiccation stress, understanding the adaptive mechanisms of foundational species like *S. muticum* becomes ecologically urgent. The identified genes, particularly those involved in endoplasmic reticulum homeostasis and signaling, could serve as biomarkers for monitoring stress resilience in natural populations. Conversely, targeting these pathways could inform strategies to control invasive *S. muticum* populations by sensitizing them to desiccation during low-tide periods. Collectively, these findings offer biotechnological insights for enhancing stress resilience in cultivated seaweeds or mitigating invasive spread through targeted pathway disruption.

### 4.1. Desiccation Stress Regulatory Genes in S. muticum

Little is known about the perception of regulatory genes in *Sargassum*. In the model plant *Arabidopsis*, drought stress signaling is classified into abscisic acid (ABA)-dependent and ABA-independent pathways [12,29,30]. ABA triggers diverse downstream responses, including the activation of kinases, phosphatases, and ubiquitin pathway proteins [14]. In the current investigation, upregulated mitogen-activated protein kinase subtypes (*MAPK1_2* and *MAPK6*) along with serine/threonine kinases *SNRK2* and *CTR1* were identified. Within the signaling hierarchy, *SNRK2* and *CTR1*, positioned upstream, function as primary transducers, while the *MAPK* isoforms act as downstream components in ABA-responsive pathways. This kinase cascade architecture suggests an integrated signaling mechanism facilitating ABA-mediated osmotic adjustment, thereby augmenting stress resilience in *S. muticum*. These kinases may also regulate the expression of stress-responsive genes [14]. The absence of detectable canonical ABA biosynthetic genes in our transcriptome raises intriguing questions. While *SNRK2* and *CTR1* were upregulated, the signaling ligands triggering this cascade remain elusive. Additionally, plants synthesize ABA under drought conditions, inducing stomatal closure to minimize water loss [3], a process mediated by ABA-dependent Ca^2^⁺ signaling [31]. Calmodulin (*CALM*), a critical Ca^2^⁺ sensor in plants, relays these signals to maintain homeostasis and thus facilitate drought adaptation [32,33,34]. The upregulation of calmodulin genes observed here implies that intracellular Ca^2^⁺ signaling is integral to desiccation adaptation in *S. muticum*. However, limited research on desiccation stress in brown seaweeds, combined with our preliminary findings, precludes the delineation of a complete regulatory network. Thus, it remains uncertain whether *Sargassum* employs regulatory mechanisms analogous to those of higher green plants.

### 4.2. Desiccation Stress Functional Genes in S. muticum

Functional genes typically encode proteins that directly counteract environmental stress [3]. In *S. muticum*, these include *HSPs* and endoplasmic reticulum homeostasis-related genes. HSPs, conserved molecular chaperones, promote protein refolding and prevent the accumulation of misfolded proteins under stress [35]. Studies in higher plants, such as barley [36], rice [37], maize [38], and *Arabidopsis* [39], report the induction of *HSP17*, *HSP22*, *HSP70*, and *HSP90* under drought conditions. Similarly, *HSP40* is upregulated in the red alga *Pyropia tenera* during desiccation, underscoring the universal role of molecular chaperones in mitigating protein denaturation under stress [14]. Here, we observed the differential upregulation of *HSP20*, *HSP40*, and *HSP70*, suggesting shared desiccation adaptation mechanisms between macroalgae and higher plants. Significant upregulation was observed in key protein homeostasis regulators, including molecular chaperones (*CALR* and *CANX*) and the folding catalyst *PDIA6*, under desiccation conditions (Table S3). Bioinformatic analyses localized these stress-responsive genes to the endoplasmic reticulum protein processing pathway, where they are known to maintain proteostatic control [35,40,41]. Mechanistically, their gene products orchestrate cellular recovery through the coordination of the unfolded protein response and targeted degradation pathways—processes evolutionarily conserved in photosynthetic eukaryotes [10,13]. In terrestrial plants, endoplasmic reticulum stress responses are typically linked to drought-induced protein misfolding, activating the unfolded protein response to restore proteostasis [36,37,38,39]. Our data suggest that *S. muticum* employs a similar strategy, but with distinct regulatory nuances. For instance, the coordinated upregulation of *PDIA6*—a disulfide isomerase—implies enhanced redox regulation during dehydration, a feature less emphasized in green algae [14]. This may reflect the evolutionary divergence of brown algae, which possess complex secondary metabolites (e.g., fucoxanthin and phlorotannins) that could interact with endoplasmic reticulum stress pathways to buffer oxidative damage [14,42,43]. Concurrently, transcriptomic data revealed a pronounced upregulation of tubulin-encoding genes, suggesting cytoskeletal involvement in osmotic stress adaptation [44,45,46,47]. Such cytoskeletal remodeling may serve dual roles in microtubule reorganization and cellular volume regulation, potentially preserving membrane integrity during rehydration cycles [45]. These collective findings highlight the critical importance of multi-layered homeostatic regulation in *S. muticum*’s desiccation resilience.

Notwithstanding these insights, conventional bulk RNA-seq analyses using mixed cell populations inherently mask tissue-specific expression patterns. Future investigations employing spatial transcriptomic approaches could decipher localized molecular adaptations across thallus regions, while single-cell resolution studies may unveil specialized cellular strategies. Such methodological advancements promise to elucidate the ecophysiological mechanisms underlying the success of intertidal macroalgae.

While this study captures the transcriptomic response after a 6 h desiccation period, it reflects an endpoint rather than the dynamic progression of desiccation stress. To more comprehensively understand the molecular mechanisms of desiccation responses, future studies could explore the kinetics and sequence of events during the process. For example, sampling at multiple timepoints (e.g., 0, 2, 4, and 6 h) could reveal how gene expression and metabolic changes unfold over time, and identify master regulators of the adaptation cascade, providing deeper insights into the adaptation process.

## 5. Conclusions

*S*. *muticum*, an intertidal seaweed, has evolved mechanisms to withstand desiccation stress. By comparing transcriptomes from *S. muticum* under control and desiccation conditions, we identified differentially expressed genes (DEGs) categorized into regulatory genes (e.g., mitogen-activated protein kinase and calmodulin) and functional genes (e.g., heat shock proteins and tubulin). By integrating regulatory kinases, molecular chaperones, and metabolic reprogramming, this species orchestrates a multifaceted response to desiccation stress. GO enrichment analysis indicated that upregulated genes were primarily associated with metabolic processes, notably glutathione metabolism, while downregulated genes were linked to transport functions, such as ammonium transport. KEGG analysis highlighted “protein processing in endoplasmic reticulum” and “MAPK signaling pathway-plant” as the most enriched pathways, indicating extensive biological adjustments to desiccation. This study delineates core molecular mechanisms underlying desiccation adaptation in *S. muticum*, establishing the conceptual framework for investigating post-stress recovery dynamics and persistent tolerance acquisition. The findings not only advance our understanding of algal stress biology but also hold translational potential. Future studies should explore temporal dynamics of gene expression during dehydration–rehydration cycles to identify master regulators of stress recovery.

## Figures and Tables

**Table 1:** Primer sequences for the genes used for qRT-PCR.

Gene Name	Description	Primer Sequences (5′-3′)
*TUBA*	tubulin alpha	F: CAACACCACCGCTATTGCTGAG R: TTCCTCCATACCTTCACCTACATACC
*HSP70*	heat shock 70	F: CTCGTGGTGTGCCTCAAATCG R: TGTGATGGTTATCTTGTTCTCCTTGC
*EEF1A*	elongation factor 1-alpha	F: CTTGACGCTATCTTGCCACCTTC R: ATTCCAGTTTCAACACGACCTACAG
*HSP20*	HSP20 family protein	F: TTCTTCTCTCCATCGCCATTCTTTG R: CGTATCTTCAACTGTAGGGATCAAGG
*CALM*	calmodulin	F: AGGACGGGAACGGGAACATC R: GTGTCAACCTTCGCCATCATCTC
*PDIA6*	protein disulfide-isomerase A6	F: TTCCATTATGACGCCTGTGATTCG R: CGGTTTGATTGTCTGTCGCATTC
*COX3*	cytochrome c oxidase subunit 3	F: TTCTCTATTGCTGACAGGGTTTATGG R: TTGCTCCAACCAGTACATGAAGTC
*GAPDH*	glyceraldehyde 3-phosphate dehydrogenase	F: CCAAGGCTGTCGGTAAAGTCATTC R: ACGGTTAAGTCAACAACGGATACG
*SNRK2*	serine/threonine-protein kinase SRK2	F: AAGTGACCAGGCAGGAACCAG R: GCAGCGACCACAATCAATACTCC
*ABCC1*	ATP-binding cassette, subfamily C (CFTR/MRP), member 1	F: GACGATGGCGGAGCAGGAG R: TGGAGACGGTACGAGGCATTG
*actin*	actin	F: TTGATCTGTTGAGTTACCTGAGTTGG R: GTTACCGATGGCGTTCACTACTG

**Table 2:** Summary of transcriptome sequencing data.

Sample	Clean Bases [G]	Clean Reads [M]	Q30 [%]	GC [%]
A1	5.30	17.21	92.81	51.58
A2	6.47	21.12	93.03	51.58
A3	6.80	22.11	92.88	51.91
B1	6.78	22.05	93.02	51.60
B2	6.63	21.52	92.90	51.79
B3	5.93	19.27	92.90	51.60

**Table 3:** Summary statistics of functional annotation of unigenes.

Database	Number of Unigenes	Percentage (%)
NR	36,687	55.43
Pfam	25,306	38.23
eggNOG	25,060	37.86
KOG	20,422	30.85
Swissprot	19,016	28.73
GO	17,939	27.10
KEGG	9558	14.44
Annotation in all databases	6969	10.53
At least one database annotation	37,950	57.33
Total number of unigenes	66,192	100

## Data Availability

All sequencing data were deposited in the NCBI Short Read Archive (SRA) database under the BioProject ID: PRJNA1249968. Relevant supporting data can be found within the article and additional files.

## Supplementary Materials

The following supporting information can be downloaded at: https://www.mdpi.com/article/10.3390/genes16050587/s1, Table S1: Annotation analyses of DEGs under desiccation stress, Table S2: KEGG pathway analyses of DEGs under desiccation stress, Table S3: DEGs enriched in the “protein processing in endoplasmic reticulum” pathway, Table S4: DEGs enriched in the “MAPK signaling pathway-plant” pathway.

## Abbreviations

The following abbreviations are used in this manuscript:

DEGs	Differential expression genes
MAPK	Mitogen-activated protein kinase
GO	Gene Ontology
KEGG	Kyoto Encyclopedia of Genes and Genomes
NR	Non-Redundant Protein Sequence Database
Pfam	Protein families database
eggNOG	Evolutionary genealogy of genes: Non-supervised Orthologous
KOG	EuKaryotic Orthologous Group
Swissprot	SwissProt Database
qRT-PCR	Quantitative Reverse Transcriptase Polymerase Chain Reaction
ATP	Adenosine triphosphate
